# Controlling nutritional status score during hospitalization as a predictor of clinical outcome in patients with aneurysmal subarachnoid hemorrhage

**DOI:** 10.1038/s41598-023-39938-1

**Published:** 2023-08-07

**Authors:** Shinya Shimizu, Tatsunori Hanai, Yusuke Egashira, Yukina Sato, Kumiko Sekiya, Shohei Nishida, Masashi Ishihara, Takuma Ishihara, Ryuta Asada, Ryo Kobayashi, Akio Suzuki

**Affiliations:** 1 Department of Pharmacy, Gifu University Hospital, 1-1 Yanagido, Gifu, 501-1194 Japan; 2 First Department of Internal Medicine, Gifu University Hospital, Gifu, Japan; 3 Department of Neurosurgery, Gifu University Graduate School of Medicine, Gifu, Japan; 4 Innovative and Clinical Research Promotion Center, Gifu University Hospital, Gifu, Japan; 5 Laboratory of Advanced Medical Pharmacy, Gifu Pharmaceutical University, Gifu, Japan

**Keywords:** Predictive markers, Cerebrovascular disorders, Stroke, Biomarkers

## Abstract

Aneurysmal subarachnoid hemorrhage (aSAH) is a serious condition with high mortality and a high permanent disability rate. In this study, we examined the association of clinical outcome with the Controlling Nutritional Status (CONUT) score during hospitalization in aSAH patients. A single-center, retrospective observational study was conducted at Gifu University Hospital. Patients transported to the emergency room for aSAH and diagnosed with World Federation of Neurosurgical Societies (WFNS) grade III and IV aSAH between April 2004 and March 2021 were enrolled. A logistic regression model was constructed to evaluate the association of the CONUT score with a modified Rankin scale (mRS) ≥ 3 and delayed cerebral ischemia (DCI). 127 patients diagnosed with WFNS grade III and IV aSAH were analyzed. CONUT score was significantly associated with mRS ≥ 3 during hospitalization. The score obtained by subtracting the CONUT score at admission from the maximum CONUT score was significantly associated with mRS ≥ 3 at discharge. Moreover, the score obtained by subtracting the CONUT score at admission from the maximum CONUT score during the first 14 days was significantly associated with DCI within 14 days from admission. These findings indicate that CONUT score during hospitalization may be a useful daily marker for predicting poor outcomes in aSAH patients.

## Introduction

Spontaneous subarachnoid hemorrhage (SAH) affects individuals with a mean age of 55 years and accounts for 5% of stroke cases. About 85% of SAHs are aneurysmal and 10% are non-aneurysmal perimesencephalic. Incidence of SAH is particularly high in East Asian countries, including Japan^1^. Aneurysmal subarachnoid hemorrhage (aSAH) is a serious condition with high mortality and a high permanent disability rate. The 30-day and 6-month mortality rate is 39% and 49%, respectively, and only 23% of patients have a favorable outcome, as evaluated using a modified Rankin scale (mRS) score of 0–2^2^. The social and economic losses of patients with aSAH in the event of death or inability to return to society are therefore substantial^3^.

It well known that energy expenditure in patients with SAH dramatically increases after disease onset^4–6^. Several studies have reported that SAH patients’ nutritional status on admission^7–9^ and during hospitalization^10,11^ influences their clinical outcomes. Badjatia et al. reported in a prospective study of 58 SAH patients that a negative energy balance during the first 7 days after SAH correlated with the number of infectious complications^10^. These researchers also showed in a prospective observational study of 229 SAH patients that a negative nitrogen balance during the first 14 post-bleed days is a risk factor for infectious complications and is associated with poor outcomes (mRS ≥ 4) at 3 months^11^. Thus, it is important to identify a useful marker of the nutritional status of SAH patients during hospitalization.

The controlling nutritional status (CONUT) score, which is calculated based on patients’ serum albumin, total lymphocytes and total cholesterol, is associated with Full Nutritional Assessment (FNA) and was recently introduced as a nutritional screening tool^12^. Qi et al. demonstrated in a retrospectively analysis of 252 aSAH patients that a CONUT score < 4 at admission independently predicted patients’ functional outcome status at 3 months after aSAH^7^. However, it is unclear whether the CONUT score during hospitalization influences the functional outcomes of aSAH patients.

Here, we retrospectively analyzed the relationship between clinical outcomes and the CONUT score in aSAH patients during hospitalization.

## Results

### Patient demographics

**Figure 1:**
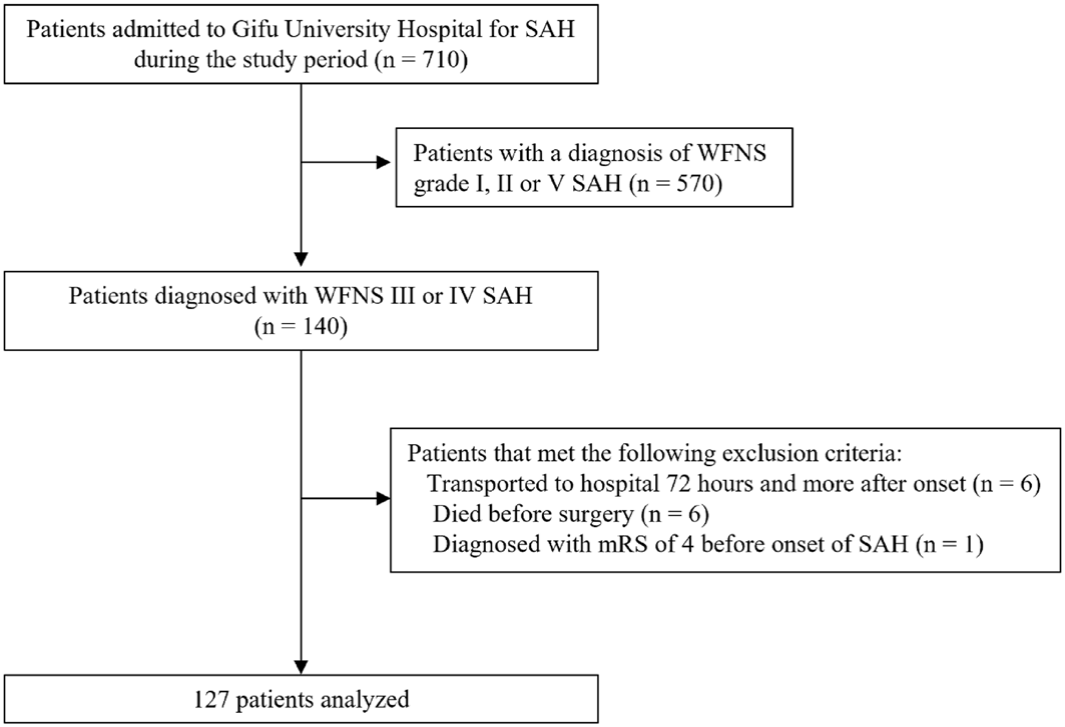
CONSORT diagram. WFNS: World Federation of Neurosurgical Societies, SAH: subarachnoid hemorrhage.

A flow diagram of patient recruitment is shown in Fig. 1. A total of 710 patients were admitted to our hospital with SAH during the study period. Of these, the number diagnosed with WFNS grade I, grade II, grade III, grade IV and grade V SAH were 252 (35.5%), 135 (19.0%), 17 (2.4%), 123 (17.3%) and 183 (25.8%), respectively. A total of 140 patients diagnosed with WFNS grade III and IV aSAH were enrolled in this study. Among these, 13 patients were excluded because they were transported to hospital 72 h and more after onset (n = 6), died before surgery (n = 6) or had an mRS of 4 before SAH onset (n = 1). This analysis therefore included 127 patients, of whom 16 and 111 patients were diagnosed with WFNS grade III and IV SAH, respectively.

Table 1 shows the patient’s demographics. Among the 127 patients, 41 (32.3%) were male and 86 (67.7%) were female, median age was 67 years (range: 37–89), and median body mass index was 22.0 kg/m^2^ (IQR: 19.3–24.1). The median CONUT score, ALBI score, C-reactive protein/albumin ratio and mRS were 2 (IQR: 1–3), − 2.77 (IQR: − 3.08 to − 2.49), 0.043 (IQR: 0.015–0.161) and 5 (IQR: 5–5), respectively.

**Table 1:** Patient demographics.

Age, median (mini‒max)	67	(37‒89)
Sex, n (%)		
Male	41	(32.3)
Female	86	(67.7)
Height, cm	156.5	(150‒162)
Body weight, kg	53	(47‒63)
Body mass index, kg/m^2^	22.0	(19.3‒24.1)
Laboratory data		
ALB, g/dL	4.1	(3.8‒4.45)
AST, IU/L	27	(22‒36.5)
ALT, IU/L	17	(13‒25)
CRE, mg/dL	0.57	(0.49‒0.72)
CRP, mg/dL	0.18	(0.065‒0.62)
T-Bil, mg/dL	0.7	(0.5‒0.9)
T-CHO, mg/dL	188	(168‒213.5)
TP, g/dL	6.8	(6.5‒7.2)
WBC, /mL	12,270	(10.00‒15.68)
HGB, g/dL	12.9	(11.6‒14.4)
PLT, /mL	21,700	(18.1‒25.8)
Lymphocytes, /mL	11.11	(6.68‒18.27)
CONUT score	2	(1‒3)
ALBI score	− 2.77	(− 3.08 to − 2.49)
CRP/ALB ratio	0.043	(0.015‒0.161)
mRS	5	(5‒5)

All data indicate median, 25–75th percentile unless otherwise indicated.

### SAH treatment status

Treatment comprised coiling and clipping in 72 (56.7%) and 55 (46.3%) patients, respectively. The median length of hospital stay was 39 days (IQR: 27–57). The proportion of patients with an mRS of 0, 1, 2, 3, 4, 5 and 6 at discharge was 1 (0.8%), 25 (19.7%), 28 (22.0%), 15 (11.8%), 9 (7.1%), 42 (33.1%) and 7 (5.5%), respectively.

### Relationship between mRS ≥ 3 and CONUT score during hospitalization

**Figure 2:**
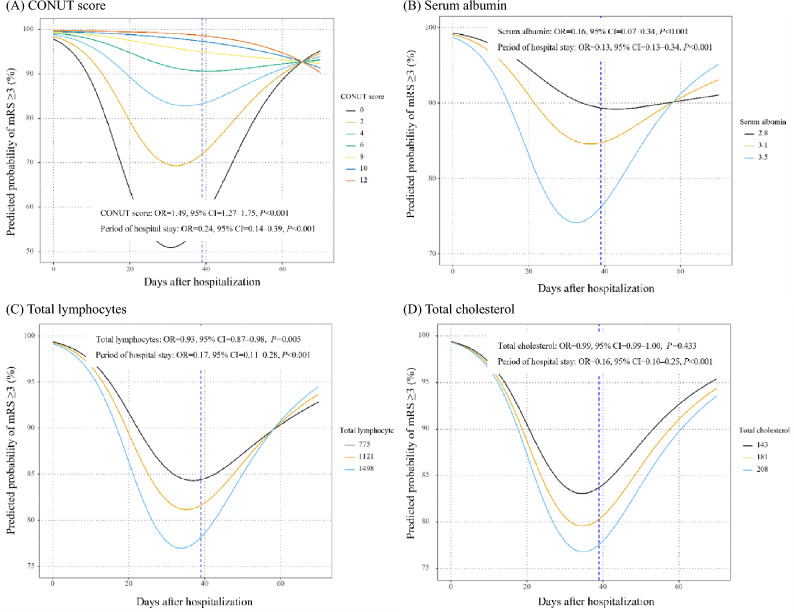
Association between modified Rankin scale (mRS) ≥ 3 and Controlling Nutritional Status (CONUT) score (**A**), serum albumin (**B**), total lymphocytes (**C**), and total cholesterol (**D**) during hospitalization.

A total of 5834 data points obtained from the 127 patients were analyzed in this study. Figure 2A depicts the association of the proportion of patients with mRS ≥ 3 with CONUT score (0, 2, 4, 6, 8, 10 and 12) from the day of admission to discharge. The proportion of patients with mRS ≥ 3 was low among those with lower CONUT scores. According to multivariable logistic regression analysis, there was a significant association of mRS ≥ 3 with CONUT score after adjustment for period of hospital stay, age and sex [odd ratio (OR) = 1.49, 95% confidence interval (CI) = 1.27‒1.75, *P* < 0.001].

Figures 2B‒D show the association of the proportion of patients with mRS ≥ 3 with the three components of the CONUT score from the day of admission to discharge. Of the three components, serum albumin and total lymphocytes, but not total cholesterol, were significantly associated with mRS ≥ 3 (serum albumin: OR = 0.16, 95% CI = 0.07‒0.34, *P* < 0.001; total lymphocytes: OR = 0.93, 95% CI = 0.87‒0.98, *P* = 0.005; total cholesterol: OR = 0.99, 95% CI = 0.99‒1.00, *P* = 0.433; Fig. 2B–D).

**Figure 3:**
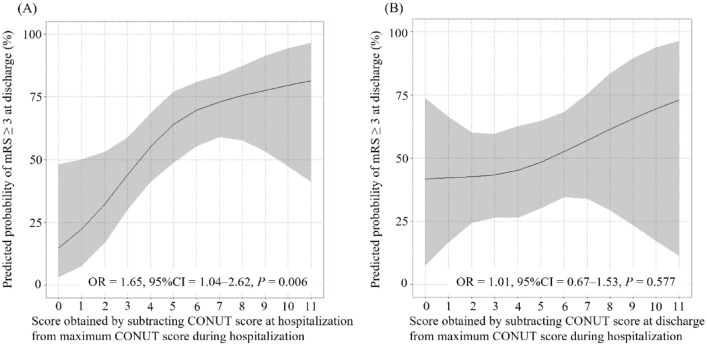
Association between the predicted probability of mRS ≥ 3 at discharge and the score obtained by subtracting CONUT score at admission from maximum CONUT score during hospitalization (**A**) and the score obtained by subtracting CONUT score at discharge from maximum CONUT score during hospitalization.

Moreover, the score obtained by subtracting the CONUT score at admission from the maximum CONUT score was significantly association with mRS ≥ 3 at discharge in the multivariable logistic regression analysis after adjusting for age and sex (OR = 1.65, 95%CI = 1.04‒2.62, *P* = 0.006, Fig. 3A). In contrast, the score obtained by subtracting the CONUT score at discharge from the maximum CONUT score was not significantly associated with mRS ≥ 3 at discharge (OR = 1.01, 95% CI = 0.67‒1.53, *P* = 0.577; Fig. 3B).

### Relationship between DCI and CONUT score during hospitalization

**Figure 4:**
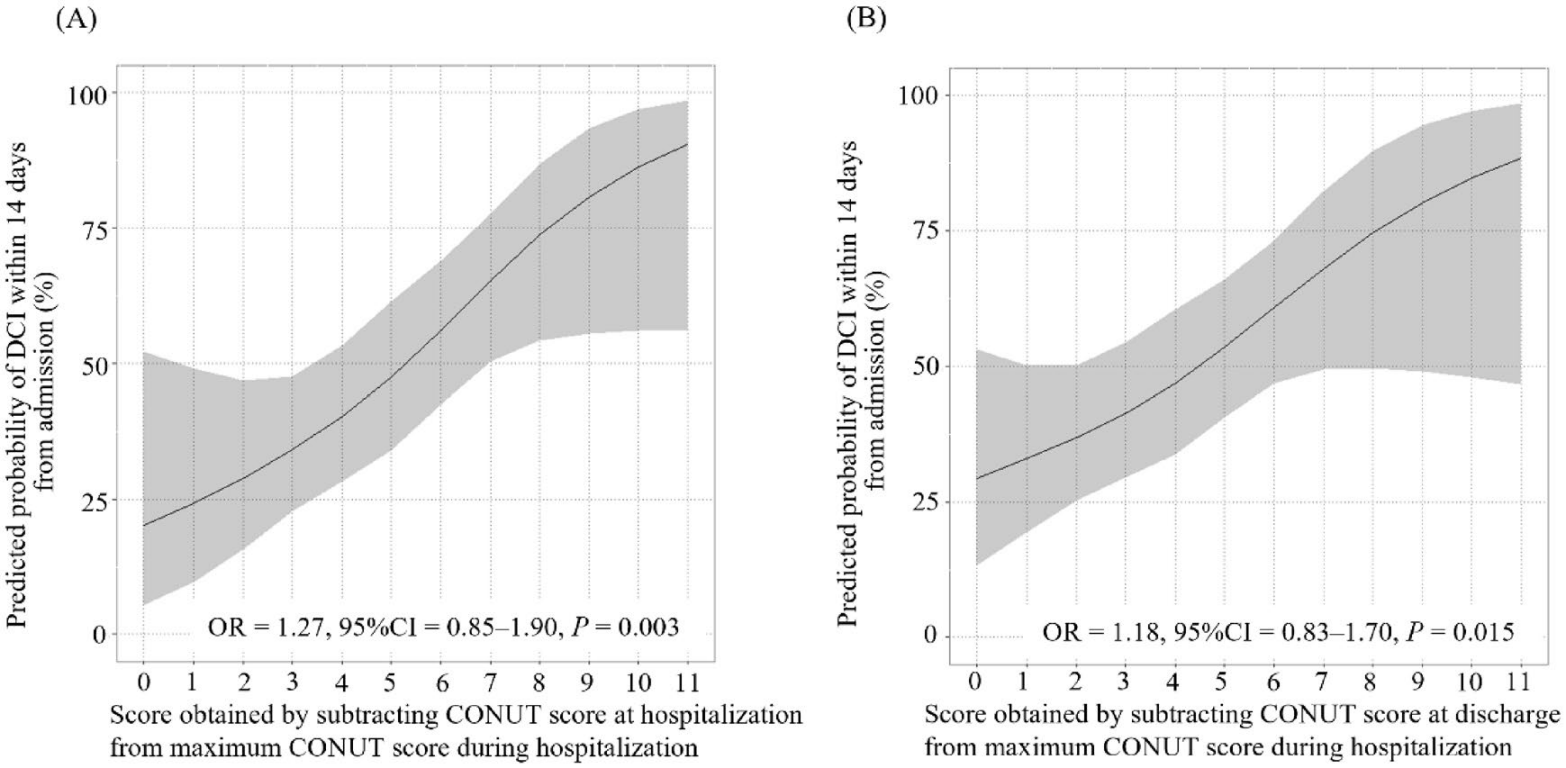
Association between the predicted probability of DCI within 14 days from admission and the score obtained by subtracting CONUT score at admission from maximum CONUT score during the 14 days from admission (**A**) and the score obtained by subtracting CONUT score at Day 14 from maximum CONUT score during the 14 days from hospitalization.

To assess the association of DCI within 14 days from admission with the CONUT score at admission, multivariable logistic regression analysis was performed with adjustment for age and sex. DCI within 14 days from admission was significantly associated with the score obtained by subtracting the CONUT score at admission from the maximum CONUT score during the first 14 days (OR = 1.27, 95% CI = 0.85‒1.90, *P* = 0.003, Fig. 4A) and the score obtained by subtracting the CONUT score on Day 14 from the maximum CONUT score during the first 14 days (OR = 1.18, 95% CI = 0.83‒1.70, *P* = 0.015; Fig. 4B).

### Relationship between mRS ≥ 3 and DCI and the CONUT score at admission

**Figure 5:**
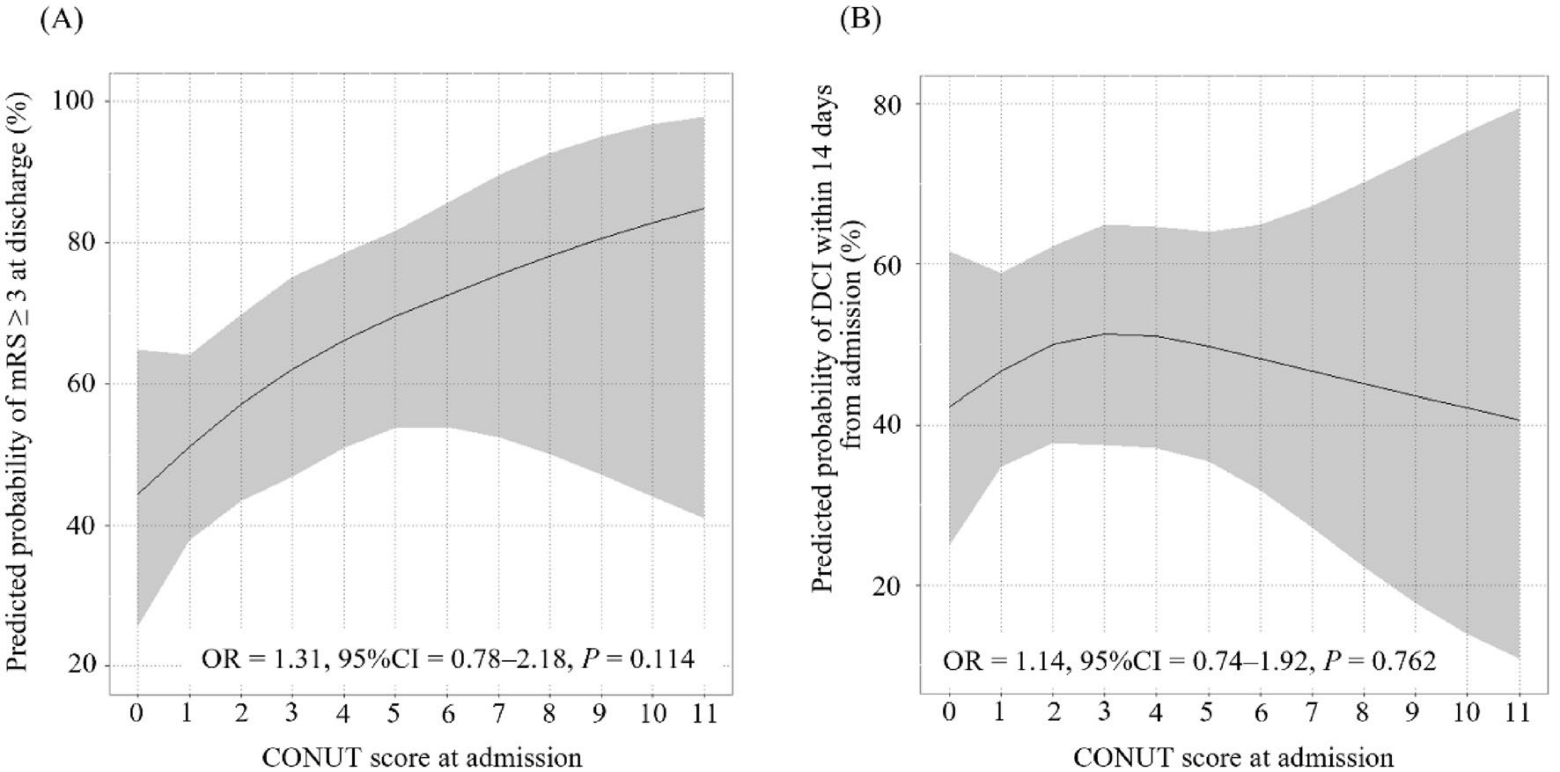
Association between CONUT score at admission and mRS ≥ 3 at discharge (**A**), and DCI within 14 days from admission (**B**).

According to multivariable logistic regression analysis adjusted for age and sex, mRS ≥ 3 at discharge and DCI within 14 days from admission were not significantly associated with CONUT score at admission (mRS: OR = 1.31, 95% CI = 0.78‒2.18, *P* = 0.114; Fig. 5A; DCI: OR = 1.19, 95% CI = 0.74‒1.92, *P* = 0.762; Fig. 5B).

## Discussion

In this retrospective study, we identified a significant association between mRS ≥ 3 and CONUT score during hospitalization in aSAH patients. The score obtained by subtracting the CONUT score on hospitalization from the maximum CONUT score during hospitalization was also significantly associated with the proportion of patients with mRS ≥ 3 at discharge. Additionally, the incidence of DCI during the first 14 days was significantly associated with the score obtained by subtracting the CONUT score during hospitalization from maximum CONUT score during the first 14 days. To our knowledge, this is first report to show an association between a change in CONUT score during hospitalization and clinical outcome in aSAH patients.

The CONUT score was developed to screen for undernutrition in hospital populations^12^. Numerous studies have reported an association between short- and/or long-term clinical outcomes and CONUT score in various acute diseases, including heart failure^13,14^, coronary syndrome^15^, myocardial infarction^16^, pulmonary embolism^17^ and ischemic and hemorrhagic stroke^7,18,19^. In contrast, few reports have examined the association of changes in CONUT score during hospitalization with clinical outcomes. An observational study by Takada et al. that included 1705 hospitalized patients with heart failure showed that changes in CONUT score during hospitalization were associated with cardiovascular death and readmission for heart failure^20^. In the present study, multivariable logistic regression analysis adjusted for period of hospital stay, age and sex revealed a significant association between CONUT score and mRS ≥ 3 during hospitalization, and the proportion of patients with mRS ≥ 3 was low among those with lower CONUT scores during hospitalization. Additionally, the scores obtained by subtracting the CONUT score at admission from the maximum CONUT score and maximum CONUT score during the first 14 days were significantly associated with mRS ≥ 3 and DCI during the first 14 days. These findings indicate that CONUT score during hospitalization might be a useful predictor of clinical outcome in aSAH patients, and that an increase in CONUT score during hospitalization may be a risk factor for worse outcomes in these patients. Thus, early appropriate nutritional management, which improves CONUT score, during hospitalization might lead to improved clinical outcomes in aSAH patients.

The CONUT score reflects the body’s immune-nutritional status, and serum albumin levels, total lymphocyte counts, and total cholesterol content are markers of the body's nutritional status, immune function, and lipid metabolism parameters, respectively^12^. Of the components of CONUT, serum albumin and total lymphocytes were significantly associated with mRS ≥ 3 in this study. Consistent with our results, previous studies also reported an association of clinical outcomes with serum albumin^21–23^ and lymphocytes^24,25^. Poor nutritional status during hospitalization is associated with poor outcomes in SAH patients^4–6^. Additionally, it is well understood that immune inflammation in the central nervous system is an important mechanism of early brain injury after aSAH and plays a critical role in the development of cerebral vasospasm and DCI^26^. Moreover, several studies have reported the involvement of serum free fatty acid levels and the effectiveness of supplementation with omega-3 fatty acids for preventing complications such as DCI after SAH^4,27,28^. Omega-3 fatty acids have numerous pharmacologic actions, including anti-inflammatory activity, serum lipid-lowering effects and suppression of platelet aggregation. These previous findings indicate that, in addition to nutritional status, monitoring of immune status may also be important to preventing complications such as DCI after aSAH. Accordingly, CONUT score may a better marker of aSAH outcome than serum albumin and lymphocytes alone.

In the present study, mRS ≥ 3 at discharge and DCI within 14 days from admission were not significantly associated with CONUT score at admission, which is inconsistent with a previous report by Qi et al^7^. This discrepancy may be related to the difference in study populations: the present study enrolled patients with more severe SAH (WFNS grade III and IV) than that by Qi et al. Kasuya et al. also reported that the elevation of resting energy expenditure and the nitrogen deficits in patients with grade III, IV or V were significantly higher than in patients with grade I or II^4^. In critically ill patients, nutritional status during hospitalization may play a greater role in clinical outcome than nutritional status at onset. It is well known that, as with traumatic brain injury, patients with SAH display hypermetabolism, increased catabolism, nitrogen loss, insulin resistance in brain and elevation of resting energy expenditure^29,30^. Surgical clipping after SAH induces a catabolic state with significantly elevated resting energy expenditure and nitrogen deficit. Similarly, SAH patients treated with endovascular coiling were found to have significantly elevated resting energy expenditure^4,31^. Although low caloric intake and negative nitrogen balance during hospitalization are associated with a worse clinical outcome of SAH^10,11^, no optimal nutritional management for patients with SAH has yet been established. The present results may show that the implementation of nutritional management with monitoring of CONUT score during hospitalization is useful, and may lead to the establishment of optimal nutritional management in patients with SAH.

This study has several limitations. First, we were unable to evaluate the daily amount of calories or contents consumed by patient. The adequacy of the daily amounts of calories or nutritional content balances required to decrease CONUT score therefore remain unknown. Revealing these will in turn lead to revealing the underlying mechanism of improving patient outcomes after SAH. Second, the sample size was small and data were obtained from a single institution. Several factors associated with clinical outcome in patients with SAH have been reported^32,33^. For example, endovascular coiling for aSAH is associated with higher 14-day case fatality and shorter length of hospital stay than surgical clipping for aSAH^33,34^. Additionally, surgical clipping is associated with a higher rate of discharge to rehabilitation in elderly patients with SAH^35^. Despite these several factors, confounding factors in all logistic regression models of this study examined to date have been limited to two factors—age and gender—to avoid overfitting for the small sample size. Additionally, it is important to note the generalizability of the present results, give that it is impossible to avoid the selection bias occurring in a single-institution studies. Finally, due to the retrospective nature of this study, potentially relevant confounders other than age and sex may have been missed. Thus, further large-scale prospective multicenter research is needed to confirm these findings and explore the underlying mechanisms.

These limitations notwithstanding, this study showed that the CONUT score during hospitalization may be a useful daily marker for predicting poor outcomes in aSAH patients. Additionally, an increase in CONUT score after the onset of SAH may be an important risk factor for poor outcomes in aSAH patients. Further large-scale studies are warranted to verify the usefulness of the CONUT score for predicting clinical outcomes.

## Methods

### Study design and patients

We conducted a single-center, retrospective study at Gifu University Hospital. Study participants included aSAH patients who were diagnosed with World Federation of Neurosurgical Societies (WFNS) grade III and IV aSAH between April 2004 and March 2021. Patients aged ≤ 16 y, patients who were transported ≥ 72 h from aneurysm rupture, patients whose aneurysm rupture date was unknown, patients whose SAH was induced by a cause other than aneurysm rupture, patients with mRS ≥ 4 from before aneurysm rupture, and patients who died before surgery for aSAH were excluded.

### Treatment for aSAH

In this study, all subjects were treated based on the clinical practice guidelines for aSAH^36^. All patients with WFNS grade III and IV aSAH received 30 mg fasudil three times a day in the 14 days after surgery for aSAH, with dual antiplatelet therapy to prevent arterial thromboembolism implemented for one month, as appropriate. Intrathecal infusion of 60,000 units of urokinase for 3 days after coiling or clipping was usually performed to wash out the SAH. A ventriculoperitoneal shunt was placed for hydrocephalus.

### Evaluation of clinical outcomes

All patients who suffered from aSAH were graded according to the WFNS^37^ by physicians (a neurologist or neurosurgeon) at admission. The mRS^38^ was used to assess neurological function, and was evaluated by physicians every day from admission to discharge. An mRS score ≥ 3 was used to indicate poor clinical outcomes in this study. The outcome in this study was mRS, and the primary outcome was a binary variable defined as poor prognosis when mRS ≥ 3. To determine serum albumin, total lymphocytes and total cholesterol^12^, blood sampling was performed at admission, every day while the patient was in the intensive care unit and every four days while the patient was in the general ward. Delayed cerebral ischemia (DCI) was defined as a decrease in Glasgow coma scale (GCS) score of ≥ 2 points from the day to next day^39^. GCS was determined through daily monitoring by physicians.

### Data collection

The following data obtained during hospitalization were extracted from patients’ electronic medical records at the central database of our hospital and retrospectively analyzed: age, sex, height, weight, body mass index, serum albumin, alanine aminotransferase, aspartate aminotransferase, serum creatinine, C-reactive protein, total bilirubin, total cholesterol, total protein, white blood cell count, hemoglobin, platelet count, lymphocyte count, WFNS, mRS, GCS, time of aneurysm rupture, patient outcome (death), and cause of aneurysm rupture. Age, sex, height, weight, body mass index, and lymphocyte count in each patient were outputted from electronic medical records to the analysis sheet. The value of WFNS, mRS and GCS and the time of aneurysm rupture, patient outcome (death), and cause of aneurysm rupture were confirmed from the physician records in electronic medical records and posted to the analysis sheet by the researchers.

Creatinine clearance was calculated using the Cockcroft-Gault equation^40^, and the albumin-bilirubin (ALBI) score^41^ was calculated using the following equation: ALBI-score = log_10_Bilirubin (mmol/L) × 0.66 + Albumin (g/L) × (− 0.085).

### Statistical analysis

Baseline characteristics are described as median with interquartile range (IQR) for continuous variables, and frequency and percentage for categorical variables. Logistic regression models were used to confirm the association of CONUT score, serum albumin, total lymphocytes, and cholesterol with mRS ≥ 3 over with the course of hospitalization. To address repeated data, Huber-White sandwich variance estimators were used to calculate covariance. Each model included one component of the CONUT score (serum albumin, total lymphocytes, or total cholesterol) and days after hospitalization and their interaction terms. Age and gender are common patient characteristics and are thought to be factors associated with mRS and/or CONUT score^32,42^. Multicollinearity was checked using the variance inflation factor (VIF) and the model was fixed based on the fact that all values were less than 0.2. Associations of DCI within 14 days from admission and mRS ≥ 3 at discharge with the maximum CONUT score at discharge were confirmed using logistic regression models adjusted for age and sex. The association of DCI within 14 days from admission and mRS ≥ 3 at discharge with CONUT score at admission was confirmed using the same logistic regression model.

In all logistic regression models, nonlinear relationships between outcomes and explanatory variables were assessed using restricted cubic spline curves. A two-sided *p*-value less than 0.05 was considered statistically significant. All analyses were performed using IBM SPSS version 22 (IBM Japan Ltd., Tokyo, Japan) and R software version 4.2.1 (www.r-project.org).

### Ethics statement

The investigation conformed with the principles outlined in the Declaration of Helsinki^43^. The present study was performed in accordance with the guideline for human studies adopted by the Medical Review Board of Gifu University Graduate School of Medicine and notified by the Japanese government. The experimental protocol was approved by the Medical Review Board of Gifu University Graduate School of Medicine (institutional review board approval number: 2021-080). Due to the retrospective nature of the study, the Medical Review Board of Gifu University Graduate School of Medicine waived the need of obtaining informed consent.

## Data Availability

The datasets used and/or analysed during the current study available from the corresponding author on reasonable request.

## Abbreviations

ALB: Albumin
AST: Aspartate aminotransferase
ALT: Alanine aminotransferase
CRE: Creatinine
CRP: C-reactive protein
T-Bil: Total bilirubin
T-CHO: Total cholesterol
TP: Total protein
WBC: White blood cells
HGB: Hemoglobin
PLT: Platelets
CONUT: Controlling nutritional status
ALBI: Assessment of albumin–bilirubin
mRS: Modified Rankin scale
